# Natural SARS-CoV-2 Infection Affects Neutralizing Activity in Saliva of Vaccinees

**DOI:** 10.3389/fimmu.2022.820250

**Published:** 2022-03-11

**Authors:** Micaela Garziano, Olga Utyro, Mariacristina Poliseno, Teresa Antonia Santantonio, Irma Saulle, Sergio Strizzi, Sergio Lo Caputo, Mario Clerici, Andrea Introini, Mara Biasin

**Affiliations:** ^1^ Laboratory of Immunobiology, Department of Biomedical and Clinical Sciences L. Sacco, University of Milan, Milan, Italy; ^2^ Laboratory of Immunology, Department of Pathophysiology and Transplantation, University of Milan, Milan, Italy; ^3^ Unit of Infectious Diseases, Department of Clinical and Experimental Medicine, University of Foggia, Foggia, Italy; ^4^ Don C. Gnocchi Foundation, Istituto di Ricovero e Cura a Carattere Scientifico (IRCCS) Foundation, Milan, Italy; ^5^ Center for Molecular Medicine, Department of Medicine Solna, Division of Infectious Diseases, Karolinska University Hospital, Karolinska Institutet, Solna, Sweden

**Keywords:** SARS-CoV-2, saliva, neutralizing activity, antibodies, variants

## Abstract

**Background:**

SARS-CoV-2 transmission mainly occurs through exposure of the upper airway mucosa to infected secretions such as saliva, which are excreted by an infected person. Thus, oral mucosal immunity plays a central role in the prevention of and early defense against SARS-CoV-2 infection. Although virus-specific antibody response has been extensively investigated in blood samples of SARS-CoV-2-infected patients and vaccinees, local humoral immunity in the oral cavity and its relationship to systemic antibody levels needs to be further addressed.

**Material and Methods:**

We fine-tuned a virus neutralization assay (vNTA) to measure the neutralizing activity (NA) of plasma and saliva samples from 20 SARS-CoV-2-infected (SI), 40 SARS-CoV-2-vaccinated (SV), and 28 SARS-CoV-2-vaccinated subjects with a history of infection (SIV) using the “wild type” SARS-CoV-2 lineage B.1 (EU) and the Delta (B.1.617.2) strains. To validate the vNTA results, the presence of neutralizing antibodies (NAbs) to the spike receptor binding domain (RBD) was evaluated with an ELISA assay.

**Results:**

NA to SARS-CoV-2 lineage B.1 (EU) was present in plasma samples from all the tested subjects, with higher titers in SIV compared to both SI and SV. Conversely, NA was detected in saliva samples from 10.3% SV, 45% SI, and 92.6% SIV, with significantly lower titers in SV compared to both SI and SIV. The detection of NAbs in saliva reflected its reduced NA in SV.

**Discussion:**

The difference in NA of plasma vs. saliva was confirmed in a vNTA where the SARS-CoV-2 B.1 and Delta strains were tested head-to-head, which also revealed a reduced NA of both specimens compared to the B.1 variant.

**Conclusions:**

The administration of SARS-CoV-2 vaccines was associated with limited virus NA in the oral cavity, as measured in saliva and in comparison to plasma. This difference was more evident in vaccinees without a history of SARS-CoV-2 infection, possibly highlighting the importance of local exposure at the site of virus acquisition to effectively prevent the infection and block its spread. Nevertheless, the presence of immune escape mutations as possibly represented by the SARS-CoV-2 Delta variant negatively affects both local and systemic efficacy of NA associated with vaccination.

## Introduction

Severe Acute Respiratory Syndrome Coronavirus 2 (SARS-CoV-2), the etiological agent of Coronavirus Disease 2019 (COVID-19) pandemic, has affected more than 250 million people, causing approximately 5 million deaths in the global population as reported by the World Health Organization (WHO, November 2021). At present, the acquisition of immunity by anti-SARS-CoV-2 vaccines represents the most promising chance to contain the COVID-19 pandemic.

The virus uses the receptor binding domain (RBD), within the spike protein, to bind the angiotensin-converting enzyme 2 (ACE2) on the surface of epithelial cells in the upper respiratory tract (1). Viral transmission may occur by asymptomatic, pre-symptomatic, and symptomatic individuals through close exposure to infected secretions such as saliva, respiratory secretions, or respiratory droplets (2, 3). Thus, SARS-CoV-2 infection mainly affects the cells of the superior airways, and the nasopharyngeal swab is the specimen of choice for diagnosis of infection. However, the virus is also able to infect and replicate in the salivary glands, which is why saliva represents a safe and non-invasive sample to detect both viral RNA and SARS-CoV-2-specific antibodies (4–7). Oral tissues, encompassing salivary glands and mucosa, may play a double function: on one side, they are sites of early infection, playing a critical role in viral spreading to the lungs or the gastrointestinal tract *via* saliva (8); at the same time, they represent the first line of defense against a plethora of pathogens as already demonstrated for other microbial-associated diseases, including pneumonia (9) and inflammatory bowel diseases (10).

The mechanisms responsible for the immunological surveillance and tolerance at this site, safeguarding tissue homeostasis, include a complex network orchestrated by dendritic cells (DCs) that process and present specific antigens to resident T cells, which in turn activate B cells producing SARS-CoV-2-specific IgA (30%) and IgG (70%) (11). The induction of a microbe-specific mucosal immunity represents an unequivocal sign of an active infection (12, 13), but whether the intramuscular administration of a vaccine is capable of triggering mucosal immunity is still a matter of debate. For example, in mice, parenteral administration of the influenza vaccine has been shown to fail to induce an effective mucosal immune response (14).

Since the beginning of SARS-CoV-2 vaccination campaign, a large part of the population has already been immunized worldwide, and the presence of neutralizing antibodies (NAbs) in the serum of vaccinated subjects has been assessed. Conversely, the neutralizing response in oral mucosa needs to be further investigated. Given the preponderance of these routes in establishing new infections, we optimized the gold standard virus neutralization assay (vNTA), requiring live pathogen and largely employed to test plasma samples (5–7, 15–17) to detect the presence of neutralizing activity (NA) in saliva samples from infected and/or vaccinated subjects.

## Methods

### Study Design

**Figure 1:**
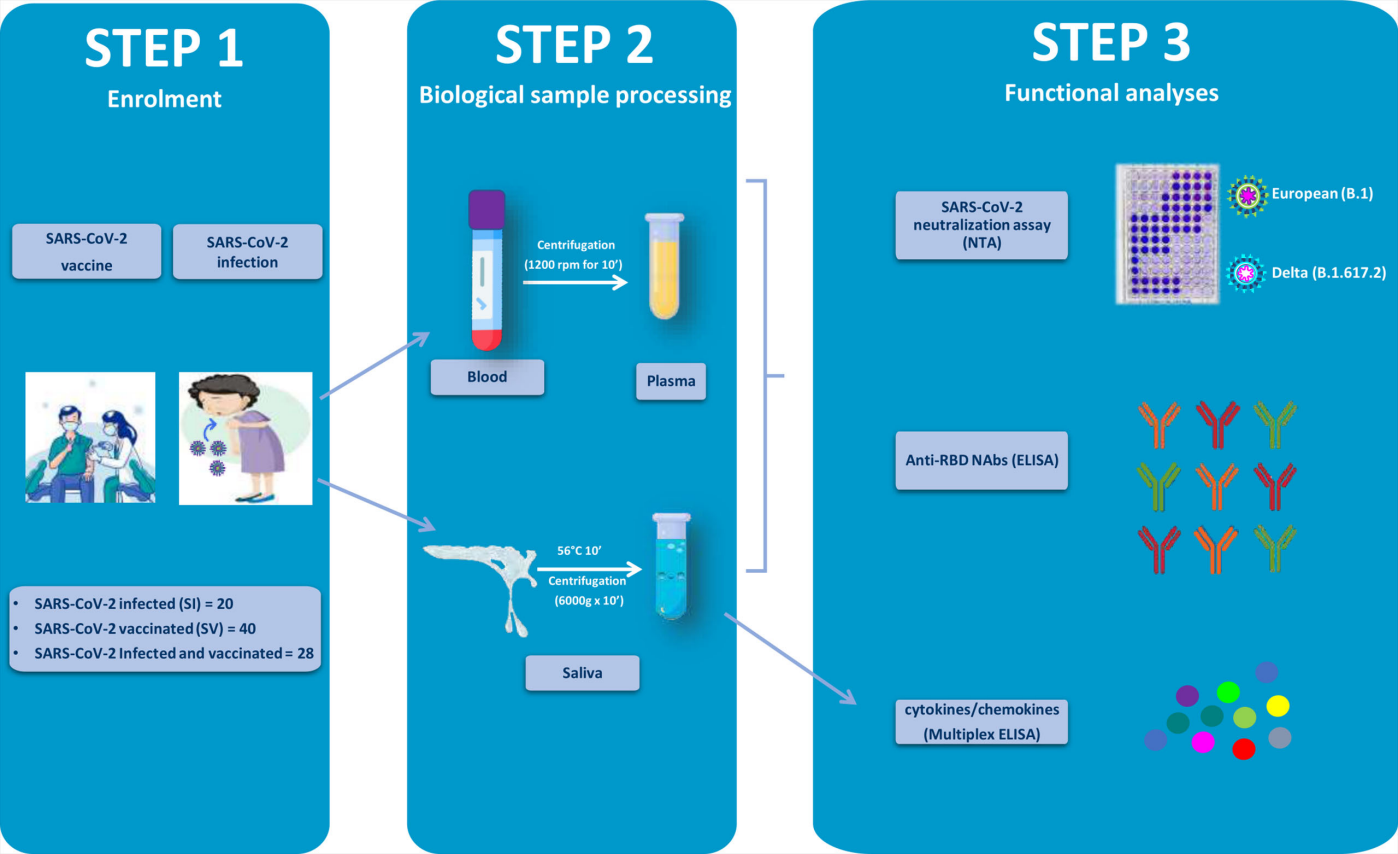
Graphical representation of the study workflow. SARS-CoV-2-infected (SI), vaccinated (SV), and infected–vaccinated (SIV) subjects were enrolled in the study (Step 1). Blood and saliva samples were collected and processed (Step 2) so as to be analyzed for neutralizing activity (NA) by SARS-CoV-2 neutralization assay (NTA), neutralizing antibodies (Nabs) by enzyme-linked immunosorbent assay (ELISA), as well as cytokine production (Multiplex ELISA).

An observational study was designed to evaluate the development of humoral immunity in SARS-CoV-2-infected (SI), SARS-CoV-2-vaccinated (SV), and SARS-CoV-2-infected and -vaccinated (SIV) subjects induced by BNT162b2 (Comirnaty) or AZD1222 anti-SARS-CoV-2 vaccines. The primary end point of the study was to optimize a vNTA in order to compare samples representative of the systemic and local response in the oral cavity to SARS-CoV-2, i.e., plasma and saliva, of the same individual, as well as between SV and SIV within each of two compartments. Secondary end points were (i) validation of the vNTA as a surrogate of the presence of SARS-CoV-2-specific NAbs in saliva, and (ii) application of the vNTA to evaluate virus NA of saliva against the currently main variant of concern, Delta. The study design is summarized in **Figure 1**.

### Virus and Cell Lines

SARS-CoV-2 variants, including the lineage B.1 (EU) (accession number: EPI_ISL_412973), assumed as comparator virus, and the Delta (lineage B.1.617.2) (accession number: EPI_ISL_1970729) were isolated from positive nasopharyngeal swabs (NPS). All the strains were identified by means of whole genome sequencing and the sequences were submitted to GISAID. The virus was propagated in VeroE6 cells (ATCC^®^ VERO C1008, CRL-1586™) and viral titers were determined by Median Tissue Culture Infectious Dose (TCID_50_) endpoint dilution assay. Briefly, serial 10-fold dilutions of viral suspension, from 10^6^ to 10^−4^ TCDI_50_/ml (50 μl), were plated onto 96-well plates, incubated at 37°C in 5% CO_2_ and checked daily to monitor the virus-induced cytopathic effect (CPE) by Optical microscope observation (ZOE™ Fluorescent Cell Imager, Bio-Rad, Hercules, CA, USA). Seventy-two hours post infection (hpi) viral titer was determined by crystal violet dyeing method, as previously described (18). All the experiments with SARS-CoV-2 virus were performed in a BSL3 facility.

### Study Population and Sample Collection

Plasma and saliva samples were obtained from 20 SARS-CoV-2 SI [mean age (years) ± DS: 29.4 ± 20.5; range: 18–83; female: 60%], 40 SARS-CoV-2 SV [mean age (years) ± SD: 34.1 ± 11.5; range: 18–62; female: 67.5%], and 28 SARS-CoV-2 SIV [mean age (years) ± SD: 41.36 ± 19.19; range: 18–61; female: 57.14%], enrolled at Infectious Diseases Unit, Policlinic “Riuniti” of Foggia (Italy). SARS-CoV-2 infection was determined by SARS-CoV-2 molecular test of nasopharyngeal swabs. All the SARS-CoV-2-infected recovered patients were asymptomatic or pauci-symptomatic. The vaccinated subjects were administered either the BNT162b2 or AZD1222 vaccine (SV: 15 AZD1222 and 25 BNT162b2; SIV: 5 AZD1222 and 23 BNT162b2). All the SV and SIV subjects were administered two doses according to the specific vaccination schedules (BNT162b2: dose II administered 21 days after dose I; AZD1222: dose II administered 90 days after dose I). Subjects who were vaccinated within 6 months from SARS-CoV-2 infection recovery received just a single vaccine dose. The administered vaccine, time from infection [mean time (months) ± SE: SI = 5.7 ± 0.5; SIV = 7.9 ± 0.7], and time from vaccination [mean time (months) ± SE: SV = 3.6 ± 0.3; SIV = 3.4 ± 0.5] are reported in **Table 1**. Plasma was obtained by centrifugation of whole blood at 1,200×*g* for 10 min and storage at −20°C until use. Plasma samples were analyzed using iFlash SARS-CoV-2 IgG and IgM (C86095G–C86095M–Shenzhen YHLO Biotech Co, Shenzhen, China) to exclude a possible ongoing asymptomatic infection since the assay targets both nucleocapsid and spike proteins. Only the subjects included in the SI and SIV groups resulted to have SARS-CoV-2 N plus S antigens (**Supplementary Table 1**).

**Table 1:** Cohort study features.

	Subject no.	Gender	Age (years)	PlasmaNAb titer		Saliva NAb titer		%Anti-RBD (ELISA)	Time from infection (months)	Time from vaccination (months)	Vaccine
				WT	Delta	WT	Delta				
**SV**	1	F	30	80	nd	–	nd	40,5	–	3	AZD1222
	2	F	27	160	nd	–	nd	33	–	3	AZD1222
	3	F	33	640	nd	–	nd	nd	–	3	AZD1222
	4	M	27	40	nd	–	nd	27	–	3	AZD1222
	5	M	24	80	nd	–	nd	nd	–	3	AZD1222
	6	F	36	160	nd	–	nd	33	–	3	AZD1222
	7	F	27	20	nd	–	nd	nd	–	6	BNT-162b2
	8	F	51	40	nd	–	nd	4,5	–	6	BNT-162b2
	9	M	57	80	nd	–	nd	nd	–	6	BNT-162b2
	10	M	39	640	nd	–	nd	18	–	3	AZD1222
	11	F	22	640	nd	–	nd	nd	–	6	BNT-162b2
	12	M	22	320	nd	–	nd	nd	–	6	BNT-162b2
	13	F	30	640	nd	–	nd	nd	–	6	BNT-162b2
	14	F	38	640	nd	–	nd	19	–	3	AZD1222
	15	M	27	640	nd	4	nd	39,5	–	3	BNT-162b2
	16	M	30	160	nd	–	nd	nd	–	3	BNT-162b2
	17	F	30	80	nd	–	nd	nd	–	3	BNT-162b2
	18	F	30	80	nd	–	nd	nd	–	3	BNT-162b2
	19	F	35	320	nd	–	nd	nd	–	6	BNT-162b2
	20	M	23	320	nd	–	nd	nd	–	6	BNT-162b2
	21	F	24	640	nd	–	nd	nd	–	6	BNT-162b2
	22	F	24	1280	nd	–	nd	nd	–	6	BNT-162b2
	23	F	24	320	nd	–	nd	nd	–	4	BNT-162b2
	24	F	18	nd	nd	–	nd	nd	–	1	BNT-162b2
	25	F	18	nd	nd	16	nd	nd	–	1	BNT-162b2
	26	F	60	640	nd	–	nd	nd	–	4	AZD1222
	27	F	40	320	nd	2	nd	40,5	–	4	BNT-162b2
	28	F	62	160	nd	–	nd	nd	–	3	AZD1222
	29	M	36	320	nd	–	nd	58	–	1	BNT-162b2
	30	M	45	2560	nd	2	nd	51	–	0.5	BNT-162b2
	31	M	38	nd	nd	–	nd	61	–	3	AZD1222
	32	F	32	nd	nd	–	nd	21,5	–	4	BNT-162b2
	33	M	37	320	320	8	2	58,5	–	0.5	BNT-162b2
	34	F	50	nd	nd	–	nd	7	–	3	BNT-162b2
	35	F	43	nd	nd	–	nd	16	–	3	AZD1222
	36	M	51	10	–	nd	nd	nd	–	4	AZD1222
	37	F	33	30	20	–	–	nd	–	4	AZD1222
	38	F	45	40	20	–	–	nd	–	4	AZD1222
	39	F	18	nd	nd	–	nd	18,5	–	3	BNT-162b2
	40	F	28	nd	nd	–	nd	27	–	4	BNT-162b2
**SI**	1	M	52	160	nd	–	nd	nd	6	–	–
	2	M	18	80	nd	–	nd	nd	6	–	–
	3	F	18	160	20	4	nd	nd	6	–	–
	4	F	18	320	40	4	nd	nd	6	–	–
	5	M	19	80	nd	2	nd	21,5	3	–	–
	6	F	80	1280	80	40	nd	nd	3	–	–
	7	M	83	800	400	80	nd	nd	3	–	–
	8	M	18	160	nd	–	nd	nd	6	–	–
	9	M	22	20	nd	–	nd	nd	6	–	–
	10	F	20	80	nd	–	nd	nd	6	–	–
	11	F	20	160	nd	2	nd	nd	6	–	–
	12	F	20	80	nd	–	nd	nd	6	–	–
	13	F	19	20	nd	–	nd	nd	4	–	–
	14	F	30	40	nd	–	nd	nd	7	–	–
	15	F	51	640	nd	2	nd	nd	3	–	–
	16	F	18	nd	nd	–	nd	nd	7	–	–
	17	M	30	160	nd	–	nd	nd	7	–	–
	18	M	18	nd	nd	2	nd	nd	5	–	–
	19	F	18	nd	nd	2	nd	nd	6	–	–
	20	F	18	nd	nd	–	nd	nd	>12	–	–
**SIV**	1	F	24	1600	nd	8	nd	nd	>12	6	BNT-162b2
	2	M	40	3200	nd	8	nd	54	3	3	BNT-162b2
	3	F	55	1600	nd	32	nd	nd	4	1	BNT-162b2
	4	F	54	3200	160	16	8	nd	>12	6	AZD1222
	5	F	61	12800	nd	32	nd	nd	>12	1	AZD1222
	6	M	47	800	nd	2	nd	28,5	>12	6	AZD1222
	7	F	25	12800	nd	64	nd	nd	4	2	BNT-162b2
	8	F	22	3200	nd	4	nd	nd	>12	6	BNT-162b2
	9	F	49	3200	800	32	2	32	>12	6	BNT-162b2
	10	M	24	800	nd	–	nd	nd	7	2	BNT-162b2
	11	M	18	nd	nd	32	nd	nd	5	0.5	BNT-162b2
	12	F	44	6400	nd	8	nd	nd	5	3	BNT-162b2
	13	M	18	nd	nd	8	nd	nd	5	1	BNT-162b2
	14	F	45	3200	nd	8	nd	nd	>12	3	AZD1222
	15	F	38	1600	nd	4	nd	nd	7	5	BNT-162b2
	16	F	61	nd	nd	2	nd	47,5	>12	8	BNT-162b2
	17	F	18	nd	nd	32	2	68	6	1	BNT-162b2
	18	M	18	3200	400	8	4	79	6	3	BNT-162b2
	19	M	83	nd	nd	128	32	90	3	1	BNT-162b2
	20	M	52	3200	800	16	8	44	6	0.5	BNT-162b2
	21	F	18	nd	nd	32	4	61	6	1	BNT-162b2
	22	F	56	nd	nd	–	nd	30	10	5	BNT-162b2
	23	F	80	nd	nd	128	32	88	3	6	BNT-162b2
	24	F	22	nd	nd	16	nd	83	6	3	BNT-162b2
	25	M	25	nd	nd	4	nd	45	6	3	BNT-162b2
	26	F	55	nd	nd	4	nd	62	6	0.5	BNT-162b2
	27	M	59	nd	nd	4	nd	64	6	4	BNT-162b2
	28	M	47	120	60	nd	nd	nd	>12	8	AZD1222

-, undetectable.

ND, Not determined.

NAb, neutralizing antibody.

RBD, receptor binding domain.

All saliva samples were collected on the same day of testing, by spitting after repeated mouth-washing with water. Saliva was incubated at 56°C for 10 min and centrifuged at 6,000×*g* for 10 min. Supernatants were used for further analyses. Participants were asked not to eat, drink, or smoke at least 30 min prior to collection.

We obtained informed written consent from all the subjects to perform the procedure and analysis, according to CARE guidelines and in compliance with the Declaration of Helsinki principles. The study was approved by the Ethics Committee of Policlinic “Riuniti” of Foggia (protocol number 49/C.E./2021).

### SARS-CoV-2 Virus Neutralization Assay

At the time of use, plasma samples were thawed at room temperature and incubated at 56°C for 30 min, to inactivate the complement proteins. Neutralization activity (NA) against SARS-CoV-2 B.1 (EU) and Delta (lineage B.1.617.2) variant by vNTA was performed as follows. Briefly, 50 μl of plasma samples, starting from a 1:10 dilution followed by serial twofold series, was transferred in two wells of 96-well microtiter plates (COSTAR, Corning Incorporated, NY 14831, USA) and mixed with 50 µl of tissue culture infecting dose 50 (TCID_50_) of SARS-CoV-2. All dilutions were made in DMEM with the addition of 1% L-Glutamine, 2% penicillin and streptomycin, and 2% fetal bovine serum. After 2 h of incubation at 37°C and 5% CO_2_, 100 µl of the mixture of the supernatant containing the plasma and virus was transferred to microplates seeded with 2 × 10^4^ VeroE6 cells for 72 h at 37°C and 5% CO_2_.

As antibody concentration is lower in saliva samples than in plasma (19), and because of saliva’s natural composition, it was necessary to set up a vNTA partially modified from the one commonly used to test plasma specimens. One-hundred microliters of saliva was seeded in a 96-well microtiter plate undiluted, and then it was diluted 1:2 in the next 6 wells. Fifty microliters of SARS-CoV-2 TCID_50_ was added to each well and incubated for 2 h at 37°C at 5% CO_2_. After incubation, 100 μl of the solution containing saliva and virus was transferred to microplates seeded with 2 × 10^4^ VeroE6 cells and incubated for 72 h at 37°C and 5% CO_2_.

At the end of incubation, cells were stained with 0.1% m/v crystal violet solution (Merck KGaA, 64271 Darmstadt, Germany) previously fixed with 4% formaldehyde 37% m/v (Merck KGaA, Darmstadt, Germany) for 20 min. Microtiter plates were then washed with PBS. Wells were scored to evaluate the degree of CPE compared to the virus control. Blue staining of wells indicated the presence of NA. Neutralizing titer corresponds to the maximum dilution with the reduction of 90% of CPE. A positive titer was equal to or greater than 1:10 or 1:2 for plasma and saliva samples, respectively. Every test included plasma control (1:10 dilution) or saliva control (undiluted), cell control (VeroE6 cells alone), and viral control (threefold series dilution).

### Anti-RDB NAb Measurement

SARS-CoV-2 anti-RDB NAbs were measured employing a commercial ELISA kit (Viazyme, Delft, Netherlands). Analyses were performed on a subgroup of SV (*n* = 18) and SIV (*n* = 15) subjects. Saliva samples were preincubated with HRP-RBD. After 30 min, they were seeded into an ACE2-coated ELISA plate to reveal the presence of anti-RBD antibodies, according to the manufacturer’s guidelines. Anti-RBD quantification [1 − (OD of sample/mean OD of negative control)] × 100% was assessed on a standard curve generated by progressive 1:10 dilutions of the positive control. According to the manufacturer instructions, results below 20% threshold were considered as negative. Undetectable samples were assigned the value 10% as the midpoint between 0 and the threshold for the purpose of statistical analysis.

### Cytokine Quantification in Saliva Samples by Multiplex ELISA

The concentration of 8 cytokines/chemokines was assessed on the saliva specimens collected from a subgroup of vaccinated subjects (SV: *n* = 19; and SIV: *n* = 21) using magnetic bead-based immunoassays (Bio-Rad, CA, USA), according to the manufacturer’s protocol *via* Bio-Plex 200 technology (Bio-Rad, CA, USA). Some of the targets resulted in having values above the normal range, and an arbitrary value of 10,000 pg/ml was assigned, while 0 pg/ml was assigned to values below the limit of detection.

### Statistical Analyses

For the study variables, medians and ranges were reported for quantitative variables, and absolute and relative frequencies were reported for categorical variables. The Student’s *t*-test and analysis of variance (ANOVA) were applied when appropriate for statistical analysis to compare variables among the analyzed groups. A *p*-value < 0.05 was set as cutoff for significance. The analyses were performed using GraphPad Prism 9.

All the procedures were carried out in accordance with the GLP guidelines adopted in our laboratories.

## Results

### Neutralizing Activity in Plasma and Saliva Samples From SARS-CoV-2-Infected and/or Vaccinees

**Figure 2:**
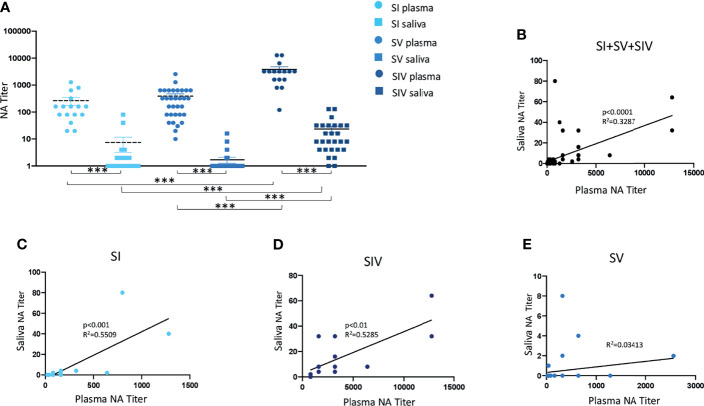
Neutralizing activity (NA) from plasma and saliva of SARS-CoV-2-infected and/or vaccinated subjects, measured by virus neutralization assay (vNTA). NA in plasma and saliva samples are reported in panel **(A)**. Correlation between NA in plasma and saliva samples of all tested subjects are showed in panel **(B)**, while correlation between NA in plasma and saliva specimens of SARS-CoV-2-infected (SI), SARS-CoV-2-infected and vaccinated (SIV), and SARS-CoV-2-vaccinated (SV) subjects are represented in panels **(C–E)**, respectively. ***p < 0.0001.

NA was not tested for plasma samples from 4 SI, 8 SV, and 12 SIV subjects, and saliva samples from 1 SV and 1 SIV because their samples were not available. Results of systemic humoral response elicited by infection and or vaccine administration showed that NA was present in 16/16 SI (100%), 32/32 SV (100%), and 16/16 SIV (100%) plasma samples. Notably, NA in plasma samples was comparable in SI (mean value ± SE: 265 ± 87.15) and SV (mean value ± SE: 388.12 ± 86.98) but significantly lower compared to SIV (mean value ± SE: 3807.5 ± 719.36) (*p* < 0.001 in both cases) (**Figure 2A**).

**Figure 3:**
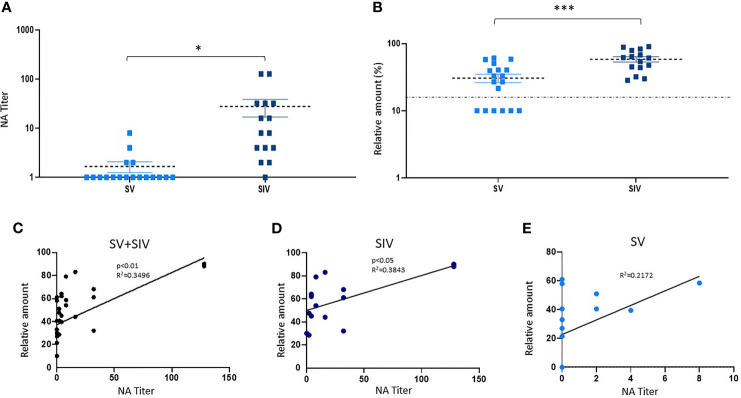
Neutralizing activity (NA) and anti-RBD NAbs titer in saliva samples from SARS-CoV-2-infected and/or vaccinated subjects. **(B)** NA quantified by vNTA in a subgroup of SV (*n* = 18) and SIV (*n* = 15) subjects is reported in panel **(A).** In the same subgroup, anti-RBD NAb production was detected by ELISA assay. The dashed line is representative of a cutoff equal to 20%. *p < 0.05, ***p < 0.001. **(C)** Taking into account all the subjects (SV+SIV), we observed a positive correlation between salivary NA tested by the two techniques (vNTA and ELISA). The production of anti-RBD NAbs quantified by ELISA was positively correlated to the NA tested by NTA in SIV **(D)** but not in the SV group **(E)**.

A different trend was observed in NA in saliva samples by vNTA. Thus, NA was present only in saliva of 5 out of 39 SV subjects (12.8%), 9/20 SI subjects (45%), and 25/27 SIV subjects (92.6%) (**Figure 2A**). In line with the results observed in plasma samples, saliva NA was significantly higher in SIV (mean value ± SE: 23.4 ± 6.48) compared to both SI (mean value ± SE: 6.9 ± 4.32) (*p* < 0.001) and SV (mean value ± SE: 0.8 ± 0.46) (*p* < 0.0001) (**Figure 3A**).

To address the potential impact of the variability in the period of time between sample collection and infection and/or vaccination, we stratified samples within each group into two subgroups according to the time of sample collection (early vs. late). Although we observed a clear decline in NA over time in both plasma and saliva samples for SI, SV, and SIV (**Supplementary Figure S1**), such change did not affect the comparison between SI and SIV, and SV vs. SIV, whose findings were replicated by analyzing samples belonging to the two identified time points separately (**Supplementary Figure S2**). Nevertheless, we were not able to validate the comparison SI vs. SV due to a substantial difference between the time from infection (approximately 6 months) and time from vaccination (approximately 3 months) for these two groups.

Of note, a superior fraction of saliva sample from SI returned a positive NA test result compared to SV in spite of such longer period of time.

By dividing SV subjects according to the vaccine they were administered, we observed that NA in plasma was higher in BNT162b2 (mean value ± SE: 487 ± 128.35)- compared to AZD1222 (mean value ± SE: 223.33 ± 74)-vaccinated subjects (*p* < 0.05) (**Supplementary Figure S3A**). Likewise, all the SV subjects who displayed a NA in saliva received the BNT162b2 vaccine (5/25 = 20%) (**Supplementary Figure S3B**).

No correlation with sex or age was detected with NA neither in plasma nor in saliva samples from the enrolled groups (data not shown).

### Correlation Between SARS-CoV-2 NA Quantified by vNTA in Plasma and Saliva Samples

NA measured by vNTA was soundly correlated in plasma and saliva samples from all of the subjects enrolled in the study (SI + SV + SIV) (*p* < 0.0001) (**Figure 2B**). By analyzing these three groups independently, we observed that such correlation was maintained for SI (*p* < 0.001) (**Figure 2C**) as well as SIV subjects (*p* < 0.01) (**Figure 2D**), but not in SV (**Figure 2E**). Moreover, by dividing SIV subjects according to the vaccine they were administered, the positive correlation between plasma and saliva NA was maintained for BNT162b2-vaccinated individuals (*p* < 0.01) (**Supplementary Figure S3C**), but not for the AZD1222 vaccines (data not shown).

### Quantification of Anti-RBD NAbs (ELISA) and Correlation With vNTA

According to the NA data obtained by vNTA on salivary samples from a subgroup of subjects (SV = 18; SIV = 15) (mean value ± SE: SV = 1.66.6 ± 0.42; SIV = 27.8 ± 11.28) (*p* < 0.05) (**Figure 3A**), the concentration of anti-RBD NAbs quantified by ELISA commercial kit was higher in SIV (mean value% ± SE: 58.40 ± 5.33) compared to SV (mean value% ± SE: 30.58 ± 4.48) (*p* < 0.0001) (**Figure 3B**). Indeed, taking into account all the subjects (SV + SIV), we observed a positive correlation between NA quantified by the two techniques (vNTA and ELISA) (*p* < 0.01) (**Figure 3C**). In particular, all of the 18 individuals who had saliva vNTA produced even anti-RBD NAbs (**Table 1**). However, of the 15 subjects who were negative for the vNTA assay, 9 (60%) tested positive to the production of anti-RBD NAbs (**Table 1**), suggesting that the two technical approaches cannot be used interchangeably because they identify different parameters.

Moreover, the production of anti-RBD NAbs quantified by ELISA was positively correlated to the production of salivary NA tested by vNTA in SIV (*p* < 0.05) (**Figure 3D**); conversely, we observed a trend towards a positive correlation, which did not reach statistical significance (*p* = 0.0512) in SV group (**Figure 3E**).

### NA in Saliva and Plasma Samples to B.1.617.2 (Delta) Strain

**Figure 4:**
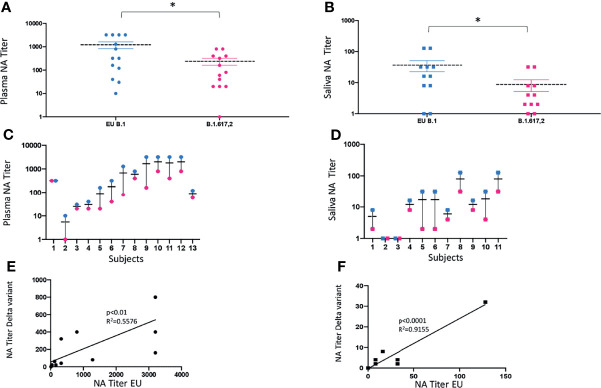
vNTA against SARS-CoV-2 lineage EU (B.1) and Delta variant (B.1.617.2). Virus neutralization assay (vNTA) titer on the Delta variant was significantly lower compared to the «wild type» SARS-CoV-2 (EU) in both plasma **(A)** and saliva **(B)** samples. *p < 0.05. Comparison between the EU variant and Delta variant in plasma and saliva samples from each enrolled subject is reported in panels **(C, D)**, respectively. Lines connect the NAbs of each individual subject. In panels **(E)** (plasma) and **(F)** (saliva), vNTA correlation between the EU and the Delta variant is described.

Saliva (*n* = 11) and plasma (*n* = 13) collected from a subgroup of subjects enrolled in the study, who displayed NA against the lineage B.1 (EU), assumed as reference virus, were tested against the Delta (lineage B.1.617.2) variant. Mean values ± SE were 1,221.5 ± 427.2 for the EU strain and 240 ± 86.7 for the Delta strain, in plasma samples (*p* < 0.05) (**Figure 4A**); and 36.4 ± 16.6 for the EU strain and 8.5 ± 4.2 for the Delta strain, in saliva specimens (*p* < 0.05) (**Figure 4B**). Thus, there was a 5-fold and 4-fold reduction in the neutralization titers against the Delta variant in plasma and saliva samples, respectively (**Figures 4C, D**), although at lower titers, NA against the Delta variant was still detectable in both biological samples from subjects who showed NA against the EU lineage. As a whole, the NA of vaccine immune sera against the EU variant was maintained to that against the Delta strain in both plasma (*p* < 0.01) (**Figure 4E**) and saliva (*p* < 0.0001) (**Figure 4F**) strain.

### Cytokine/Chemokine Quantification in Saliva Samples

In order to verify if the higher NA detected in samples from BNT162b2-vaccinated subjects was associated with an increased immune activation, we assessed the levels of 8 cytokines, including classic pro- and anti-inflammatory mediators like IL-6, IL-8, IL-10, IFNγ, and TNF, in saliva samples from SV and SIV subjects. No differences in cytokine concentration were observed by comparing saliva samples from SV and SIV, suggesting that infection does not influence the release of cytokines in the oral mucosa in response to vaccination (data not shown). However, overall cytokine production was higher in saliva specimens from BNT162b2 (SIV+SV: *n* = 28)- compared to AZD1222 (SIV+SV: *n* = 12)-vaccinated individuals with a statistically significant difference for IL-6 (*p* < 0.05), IL-10 (*p* < 0.01), and IFNγ (*p* < 0.05) (**Supplementary Figure S4**). The time of sample collection from vaccination was comparable between AZD1222 and BNT162b2 groups (mean month value ± SE: AZD1222 = 3.4 ± 0.3; BNT162b2 = 3.3 ± 0.3).

## Discussion

SARS-CoV-2 is an airborne virus that infects epithelial cells of the mucosa of the upper airways to eventually spread further, causing pulmonary and multi-organ infection and damage in some patients (20). Several studies have shown that saliva contains infectious SARS-CoV-2 particles in both symptomatic and asymptomatic individuals, and it can provide useful information on local immunity at the primary site of virus acquisition (8, 19, 21, 22). Such knowledge is of pivotal importance for the development of effective immunomodulatory strategies to prevent and cure the infection, including vaccines. Although they have been widely tested and used in humans, the outcome of SARS-CoV-2 vaccines on local virus-specific immune responses in the airway mucosa is still poorly characterized. To this end, we decided to optimize and validate a virus neutralization test, which has long been used to estimate antibody-mediated protection upon vaccination in plasma samples, to investigate the NA of saliva in a cohort of subjects with different history of SARS-CoV-2 infection and/or vaccination.

As previously documented by other authors (6, 23, 24), our current results showed that NA is present in 100% of serum samples from all the enrolled groups (SI, SV, and SIV), although the neutralization titer was significantly higher in SIV compared to both SI and SV. Conversely, NA in saliva specimens was detected in almost all SIV individuals (92%), but just in half of SI and 20% of SV and only following BNT162b2 vaccination in the latter. In line with two recent studies on virus-specific antibody detection in saliva of vaccines (25, 26), our results suggest that intramuscular SARS-CoV-2 vaccination alone elicited long-lasting (3 months post-administration) oral mucosal immunity only in a minority of subjects who received two doses, while one dose of vaccine boosted an anti-SARS-CoV-2 response in those individuals who were previously infected.

This possibly underlines the importance of local exposure at the site of virus transmission to efficiently prevent the infection and avoid its spreading, a condition that is not or is partly fulfilled by intramuscular vaccination in the absence of local pre-existing immunity. In fact, the intramuscular vaccine administration route reportedly stimulates systemic immunity, whereas intranasal or oral vaccinations trigger a local immune response also characterized by active secretion of mucosal antibodies passing into the fluids wetting the mucosa (27). Current vaccines intended to elicit local protection against viruses comprise intranasal spray (FluMist) for influenza (28) and oral drops for rotavirus (RotaTeq/Rotarix) (29, 30), polio (31), and typhoid (Vivotif) (32). The biology concerning the new SARS-CoV-2 vaccine preparations, mainly mRNA vaccines, which may stimulate distinctive kinds of antibody responses in different anatomical districts, is still largely unknown and deserves dedicated investigations. In line with previous reports (33, 34), we observed a decline in NA over time in plasma for all three groups. As expected, such decline was also observed in saliva samples. Of note, although the lack of differences in NA titers between SI and SV might be explained by a longer period of time elapsed from infection in SI (6 months) than that from vaccination in SV (3 months), a 3-fold larger fraction of SI (45%) than SV (13%) displayed NA in saliva. We cannot rule out episodes of re-exposure and/or asymptomatic infection after the reported time of diagnosis for SI. However, a possible explanation to a greater longevity or efficacy of the immune response induced by natural infection may be the persistent exposure to virus antigens associated with a sub-clinical infection as detected in the intestine of recovered COVID-19 patients up to 4 months after diagnosis (35). Nevertheless, while the time from infection was comparable between SIV and SI, the vast majority of SIV (92%) displayed a superior NA in saliva as well as plasma samples collected 3 months after vaccination, thus highlighting the efficacy of the vaccine booster regimen also in subjects recovered from COVID-19.

Even if vaccination *per se* does not result in effective and/or durable antibody responses at the site of virus transmission, other determinants of mucosal immunity, that were not evaluated in the present study, may account for local protection against SARS-CoV-2. On the other hand, some recently published papers reported a different overview of salivary antibodies in SARS-CoV-2-vaccinated subjects. Nahass et al. found IgG and IgA anti-RBD antibodies as well as NA in plasma and saliva specimens from both convalescent and mRNA-vaccinated subjects (7). Likewise, Ketas and colleagues reported that anti-S-protein IgG was present in every saliva sample from recipients of 2 mRNA vaccine doses (6). Even more recently, S1-specific IgA and IgG responses with neutralizing activity were detected in the nasal mucosa of mRNA SARS-CoV-2 vaccinees (36). Yet, it should be noted that salivary antibody detection, in these studies, was assessed by techniques other than vNTA, namely ELISA, chemiluminescent immunoassay (CLIA), flow cytometry, and pseudoviruses neutralization assays. Soon after the very early stage of the pandemic, these tests have been extensively used, allowing for faster and greater testing capacity. Although they provide useful indications, these assays do not unbiasedly evaluate the ability of a biological specimen, and/or the antibodies contained therein, to neutralize the infectivity of viral particles. In fact, RBD-binding tests account for an important fraction of NAbs, but do not quantify the NA directed against epitopes other than those commonly recognized by commercial CLIA/ELISA, such as the N-terminal domain of the spike protein (37–40). Likewise, the use of pseudoviruses could lead to incongruent results because they do not entirely recapitulate the life cycle of primary isolates. In line with this observation, in our study, the results on salivary anti-RBD NAbs quantified by ELISA were not fully mirrored by those obtained by vNTA, as some samples that did not display NA in the vNTA tested positive in the ELISA test. In support of our results, Sheikh-Mohamed and colleagues (5) recently published a study providing evidence of robust anti-Spike/RBD IgG and sIgA Ab in the saliva of vaccinated subjects, but only modest levels of neutralizing capacity in saliva specimens at 2 weeks after the second vaccine dose. Additionally, Mileto et al. did not observe a correlation between the quantity of systemic antibodies detected by CLIA assays and their NA tested by vNTA in plasma from SARS-CoV-2-vaccinated healthcare workers (24). These discrepancies suggest that SARS-CoV-2 serological tests may provide incomplete information on the protective feature of systemic or local immunity. Multiple tests addressing different mechanisms underlying humoral as well as cell-mediated virus specific immune responses are, therefore, needed to address the full extent of immunity associated with natural infection and vaccination.

The appearance of new variants of concerns (VOC), with lowered susceptibility to neutralizing antibodies, raises some worries on the possibility of evading vaccination-induced NA, as already documented on plasma samples (16, 17, 41–46). To verify if SARS-CoV-2 vaccines confer immunity in the oral cavity against mutated strains, we tested the NA of saliva and plasma samples from SV and SIV in a head-to-head comparison between B.1.617.2 (Delta) and B.1 (EU) variants in the same vNTA. In SV and SIV, the Delta strain displayed a partial immune escape in both specimens as demonstrated by lower NA titers compared to the EU lineage. However, a strong positive correlation in NA titers between the two strains confirmed the observation that existing vaccines can protect from severe disease even against potential new variants (34).

Another intriguing observation rising from this study concerns the higher protective efficacy apparently triggered by BNT162b2 compared to AZD1222. Indeed, at the systemic level, the NA was significantly higher in SV vaccinated with BNT162b2 compared to AZD1222; even more oddly, salivary NA was detected only in 5 SV subjects, all of whom received the BNT162b2 vaccine. A plausible explanation stems from a recently published paper suggesting that BNT162b2 administration elicited higher IgG and IgA titers compared to adenoviral vector AZD1222, thus providing mucosal immunity activation to prevent infection at oral and nasopharyngeal mucosa (47). The degree of immune protection offered by different vaccine types is likely associated with multiple factors, possibly reflected by variations in local immunological milieu as evidenced by our cytokine analysis. *Ad hoc* studies on larger cohorts are necessary to validate this hypothesis and pinpoint the role played by each factor in the observed response.

There are some limitations to our study: this was a non-randomized observational study, and it was not planned to investigate neither the production of the different antibody subtypes (i.e., IgA, IgG, and IgM) nor their maintenance over time post infection and/or vaccination. Also, while the main antiviral function of Ab is to neutralize virions, they may also display non-neutralizing effector functions mediated *via* their Fc fragments (i.e., Ab-dependent cellular phagocytosis, Ab-dependent cellular cytotoxicity, and Ab-dependent activation of classical complement cascade), whose activity should be monitored for completeness, as already performed in previous studies (48). Moreover, the study was not prospective, and therefore, it was limited by sample availability and suffers from some variability in specimen collection time points. To be validated, these results need more detailed, prospectively designed, and randomized studies, for instance, following the administration of a third dose. Notwithstanding, in our hands, the salivary vNTA was reliable and reproducible and offered many potential advantages: (1) saliva collection is simple, safe, non-invasive, and can be collected by any individual without the need of a phlebotomist; (2) the test is easy and relatively inexpensive using standard laboratory equipment; (3) vNTA is still considered the gold standard for determining antibody protective efficacy (15) and none of the tests developed to mimic NA, by means of anti-RBD NAbs detection, can currently replace it for the functional evaluation of antibodies (49); (4) the vNTA test may be useful to evaluate the level of cross-reactivity between vaccine antisera and variant strains that may correlate with cross-protection in the host; and (5) saliva offers a glimpse into circulating antibodies, attributed to vascular leakage from the gingival crevicular epithelium. Nonetheless, compared to other commercial techniques, vNTA requires cell culture, high biocontainment laboratories (i.e., BSL-3), more time and labor, and specific technical skills, resulting in being too cumbersome to be employed in routine testing of a large number of samples. To the best of our knowledge, this is one of the first studies to assess the NA of saliva using a vNTA and multiple variants of SARS-CoV-2. We hope that the results of this study will contribute to streamline the use of relevant samples to address local immunity at mucosal sites of interest and will highlight the importance of including such analysis for an improved estimate of the efficacy of prophylactic and therapeutic interventions.

## Data Availability Statement

The raw data supporting the conclusions of this article will be made available by the authors, without undue reservation.

## Ethics Statement

The studies involving human participants were reviewed and approved by Policlinic “Riuniti” of Foggia (protocol number 49/C.E./2021). The patients/participants provided their written informed consent to participate in this study.

## Author Contributions

Each author has approved the submitted version and agrees to be personally accountable for the author’s own contributions and for ensuring that questions related to the accuracy or integrity of any part of the work are appropriately investigated and resolved. Conceptualization: MB and MG. Subject enrolment: SC, TS, and MP. Methodology: MG, OU, SS, and IS. Formal Analysis: IS and MG. Data Curation: MG and MB. Writing—Original Draft Preparation: MG, AI, and MB. Writing—Review and Editing: MB, MC, and AI. Supervision: MB. Funding Acquisition: MB. All authors contributed to the article and approved the submitted version.

## Funding

This research was partially funded by the following grants: Bando Regione Lombardia DG Welfare cod. RL_DG-WEL20MBIAS_01; CARIPLO - EXTRABANDO E PROGETTI TER-RITORIALI cod. CAR_EXT20MBIAS_01.

## Conflict of Interest

The authors declare that the research was conducted in the absence of any commercial or financial relationships that could be construed as a potential conflict of interest.

## Publisher’s Note

All claims expressed in this article are solely those of the authors and do not necessarily represent those of their affiliated organizations, or those of the publisher, the editors and the reviewers. Any product that may be evaluated in this article, or claim that may be made by its manufacturer, is not guaranteed or endorsed by the publisher.

## Supplementary Material

Supplementary Figure 1 Neutralizing activity (NA) from plasma and saliva of SARS-CoV-2 infected and/or vaccinated subjects, measured by Neutralisation assay (NTA) over time. NA in plasma and saliva samples of SI, and SIV analyzing according to the time from infection are reported in panel **(A, B)**, respectively. **(C, D)** panels show NA in plasma and saliva of SV and SIV divided according to the time from vaccine administration. No statistically significant differences were observed in neither plasma nor saliva specimens from the enrolled groups over time.

Supplementary Figure 2 Comparison of Neutralizing activity (NA) in plasma and saliva of subjects stratified according to the time from infection and/or vaccination. NA in plasma **(A)** and saliva **(B)** specimens from SI and SIV groups sampled before and after 6 months from symptoms onset. Plasma and saliva NA of SV and SIV groups are compared respectively in panel **(C, D)** before and after 3 months from vaccine administration. Significance difference are reported into the graph: *p<0.05, **p<0.01, ***p<0.001.

Supplementary Figure 3 Neutralizing activity (NA) in plasma and saliva samples of SV and SIV subjects, measured by virus neutralisation assay (vNTA). NA in plasma and saliva samples are reported in panel **(A, B)**, respectively. Vaccinated subjects were divided according to the administrated vaccine: adenovirus-based (AZD1222) or mRNA (BNT162b2). *p<0.05, **p<0.01, ***p<0.001. In panel **(C)** the positive correlation between NA quantified in plasma and saliva samples from BNT-162b2 vaccinated subjects is shown.

Supplementary Figure 4 Cytokine quantification in saliva samples from SV and SIV subjects. Cytokine concentration in saliva samples from SV (n=19) and SIV (n=21) subjects divided according to the anti-SARS-CoV-2 vaccine they were administered (AZD1222: n°=12; BNT162b2: n°=28). Mean values ± SE are reported. *p<0.05; **p<0.01.

## Abbreviations

SI, SARS-CoV-2 infected; SV, SARS-CoV-2 vaccinated; SIV, SARS-CoV-2 infected and vaccinated; vNAT, virus neutralization assay.

## References

[1] Yang J Petitjean SJL Koehler M Zhang Q Dumitru AC Chen W. Molecular Interaction and Inhibition of SARS-CoV-2 Binding to the ACE2 Receptor. Nat Commun (2020) 11:4541. doi: 10.1038/s41467-020-18319-6 32917884 PMC7486399

[2] Zhu N Zhang D Wang W Li X Yang B Song J. A Novel Coronavirus From Patients With Pneumonia in China, 2019. N Engl J Med (2020) 382:727–33. doi: 10.1056/NEJMoa2001017 PMC7092803 31978945

[3] Zhang R Li Y Zhang AL Wang Y Molina MJ. Identifying Airborne Transmission as the Dominant Route for the Spread of COVID-19. Proc Natl Acad Sci U S A (2020) 117:14857–63. doi: 10.1073/pnas.2009637117 PMC7334447 32527856

[4] Pisanic N Randad PR Kruczynski K Manabe YC Thomas DL Pekosz A. COVID-19 Serology at Population Scale: SARS-CoV-2-Specific Antibody Responses in Saliva. J Clin Microbiol (2020) 59:e02204-20. doi: 10.1128/JCM.02204-20 33067270 PMC7771435

[5] A Mucosal Antibody Response is Induced by Intra-Muscular SARS-CoV-2 mRNA Vaccination, in: Medrxiv. Available at: https://www.medrxiv.org/content/10.1101/2021.08.01.21261297v2 (Accessed November 19, 2021).

[6] Ketas TJ Chaturbhuj D Portillo VMC Francomano E Golden E Chandrasekhar S. Antibody Responses to SARS-CoV-2 mRNA Vaccines Are Detectable in Saliva. Pathog Immun (2021) 6:116–34. doi: 10.20411/pai.v6i1.441 PMC8201795 34136730

[7] Intramuscular SARS-CoV-2 Vaccines Elicit Varying Degrees of Plasma and Salivary Antibody Responses as Compared to Natural Infection, in: Medrxiv. Available at: https://www.medrxiv.org/content/10.1101/2021.08.22.21262168v1 (Accessed November 19, 2021).

[8] Huang N Pérez P Kato T Mikami Y Okuda K Gilmore RC. SARS-CoV-2 Infection of the Oral Cavity and Saliva. Nat Med (2021) 27:892–903. doi: 10.1038/s41591-021-01296-8 33767405 PMC8240394

[9] Muhlebach MS Zorn BT Esther CR Hatch JE Murray CP Turkovic L. Initial Acquisition and Succession of the Cystic Fibrosis Lung Microbiome is Associated With Disease Progression in Infants and Preschool Children. PloS Pathog (2018) 14:e1006798. doi: 10.1371/journal.ppat.1006798 29346420 PMC5773228

[10] Kitamoto S Nagao-Kitamoto H Jiao Y Gillilland MG Hayashi A Imai J. The Intermucosal Connection Between the Mouth and Gut in Commensal Pathobiont-Driven Colitis. Cell (2020) 182:447–62.e14. doi: 10.1016/j.cell.2020.05.048 32758418 PMC7414097

[11] Moutsopoulos NM Konkel JE. Tissue-Specific Immunity at the Oral Mucosal Barrier. Trends Immunol (2018) 39:276–87. doi: 10.1016/j.it.2017.08.005 PMC5843496 28923364

[12] Badia-Boungou F Sane F Alidjinou EK Ternois M Opoko PA Haddad J. Marker of Coxsackievirus-B4 Infection in Saliva of Patients With Type 1 Diabetes. Diabetes Metab Res Rev (2017) 33:1–7. doi: 10.1002/dmrr.2916 28719027

[13] Saccoccio FM Gallagher MK Adler SP McVoy MA. Neutralizing Activity of Saliva Against Cytomegalovirus. Clin Vaccine Immunol (2011) 18:1536–42. doi: 10.1128/CVI.05128-11 PMC3165217 21795465

[14] Tomar J Patil HP Bracho G Tonnis WF Frijlink HW Petrovsky N. Advax Augments B and T Cell Responses Upon Influenza Vaccination *via* the Respiratory Tract and Enables Complete Protection of Mice Against Lethal Influenza Virus Challenge. J Control Release (2018) 288:199–211. doi: 10.1016/j.jconrel.2018.09.006 30218687 PMC7111335

[15] Matusali G Colavita F Lapa D Meschi S Bordi L Piselli P. SARS-CoV-2 Serum Neutralization Assay: A Traditional Tool for a Brand-New Virus. Viruses (2021) 13:655. doi: 10.3390/v13040655 33920222 PMC8069482

[16] Liu Y Liu J Xia H Zhang X Fontes-Garfias CR Swanson KA. Neutralizing Activity of BNT162b2-Elicited Serum. N Engl J Med (2021) 384:1466–8. doi: 10.1056/NEJMc2102017 PMC7944950 33684280

[17] Edara VV Hudson WH Xie X Ahmed R Suthar MS. Neutralizing Antibodies Against SARS-CoV-2 Variants After Infection and Vaccination. JAMA (2021) 325:1896–8. doi: 10.1001/jama.2021.4388 PMC7980146 33739374

[18] Barrow KA Rich LM Vanderwall ER Reeves SR Rathe JA White MP. Inactivation of Material From SARS-CoV-2-Infected Primary Airway Epithelial Cell Cultures. Methods Protoc (2021) 4:7. doi: 10.3390/mps4010007 33430421 PMC7839057

[19] Heinzel C Pinilla YT Elsner K Friessinger E Mordmüller B Kremsner PG. Non-Invasive Antibody Assessment in Saliva to Determine SARS-CoV-2 Exposure in Young Children. Front Immunol (2021) 12:753435. doi: 10.3389/fimmu.2021.753435 34691072 PMC8531807

[20] Harrison AG Lin T Wang P. Mechanisms of SARS-CoV-2 Transmission and Pathogenesis. Trends Immunol (2020) 41:1100–15. doi: 10.1016/j.it.2020.10.004 PMC7556779 33132005

[21] Chiang SH Tu M Cheng J Wei F Li F Chia D. Development and Validation of a Quantitative, non-Invasive, Highly Sensitive and Specific, Electrochemical Assay for Anti-SARS-CoV-2 IgG Antibodies in Saliva. PloS One (2021) 16:e0251342. doi: 10.1371/journal.pone.0251342 34197468 PMC8248704

[22] Alkharaan H Bayati S Hellström C Aleman S Olsson A Lindahl K. Persisting Salivary IgG Against SARS-CoV-2 at 9 Months After Mild COVID-19: A Complementary Approach to Population Surveys. J Infect Dis (2021) 224:407–14. doi: 10.1093/infdis/jiab256 PMC8244549 33978762

[23] Krammer F Srivastava K Alshammary H Amoako AA Awawda MH Beach KF. Antibody Responses in Seropositive Persons After a Single Dose of SARS-CoV-2 mRNA Vaccine. N Engl J Med (2021) 384:1372–4. doi: 10.1056/NEJMc2101667 PMC8008743 33691060

[24] Mileto D Fenizia C Cutrera M Gagliardi G Gigantiello A De Silvestri A. SARS-CoV-2 mRNA Vaccine BNT162b2 Triggers a Consistent Cross-Variant Humoral and Cellular Response. Emerg Microbes Infect (2021) 10(1):2235–43. doi: 10.1080/22221751.2021.2004866 PMC8648019 34749573

[25] Pinilla YT Heinzel C Caminada L-F Consolaro D Esen M Kremsner PG. SARS-CoV-2 Antibodies Are Persisting in Saliva for More Than 15 Months After Infection and Become Strongly Boosted After Vaccination. Front Immunol (2021) 12:798859. doi: 10.3389/fimmu.2021.798859 34956236 PMC8695841

[26] Azzi L Dalla Gasperina D Veronesi G Shallak M Ietto G Iovino D. Mucosal Immune Response in BNT162b2 COVID-19 Vaccine Recipients. eBioMedicine (2022) 75:103788. doi: 10.1016/j.ebiom.2021.103788 34954658 PMC8718969

[27] Weniger BG Papania MJ. Alternative Vaccine Delivery Methods. Vaccines (2008), 1357–92. doi: 10.1016/B978-1-4160-3611-1.50065-9

[28] Calzas C Chevalier C. Innovative Mucosal Vaccine Formulations Against Influenza A Virus Infections. Front Immunol (2019) 10:1605. doi: 10.3389/fimmu.2019.01605 31379823 PMC6650573

[29] Folorunso OS Sebolai OM. Overview of the Development, Impacts, and Challenges of Live-Attenuated Oral Rotavirus Vaccines. Vaccines (Basel) (2020) 8:341. doi: 10.3390/vaccines8030341 PMC7565912 32604982

[30] Soares-Weiser K Bergman H Henschke N Pitan F Cunliffe N. Vaccines for Preventing Rotavirus Diarrhoea: Vaccines in Use. Cochrane Database Syst Rev (2019) 2019:CD008521. doi: 10.1002/14651858.CD008521.pub5 PMC6434239 30912133

[31] Kirkpatrick BD Colgate ER Mychaleckyj JC Haque R Dickson DM Carmolli MP. The “Performance of Rotavirus and Oral Polio Vaccines in Developing Countries” (PROVIDE) Study: Description of Methods of an Interventional Study Designed to Explore Complex Biologic Problems. Am J Trop Med Hyg (2015) 92:744–51. doi: 10.4269/ajtmh.14-0518 PMC4385767 25711607

[32] Milligan R Paul M Richardson M Neuberger A. Vaccines for Preventing Typhoid Fever. Cochrane Database Syst Rev (2018) 2018:CD001261. doi: 10.1002/14651858.CD001261.pub4 PMC6494485 29851031

[33] Cohen KW Linderman SL Moodie Z Czartoski J Lai L Mantus G. Longitudinal Analysis Shows Durable and Broad Immune Memory After SARS-CoV-2 Infection With Persisting Antibody Responses and Memory B and T Cells. Cell Rep Med (2021) 2:100354. doi: 10.1016/j.xcrm.2021.100354 34250512 PMC8253687

[34] Dupont L Snell LB Graham C Seow J Merrick B Lechmere T. Neutralizing Antibody Activity in Convalescent Sera From Infection in Humans With SARS-CoV-2 and Variants of Concern. Nat Microbiol (2021) 6:1433–42. doi: 10.1038/s41564-021-00974-0 PMC8556155 34654917

[35] Gaebler C Wang Z Lorenzi JCC Muecksch F Finkin S Tokuyama M. Evolution of Antibody Immunity to SARS-CoV-2. Nature (2021) 591:639–44. doi: 10.1038/s41586-021-03207-w PMC8221082 33461210

[36] Frontiers. The Mucosal and Serological Immune Responses to the Novel Coronavirus (SARS-CoV-2) Vaccines, in: Immunology (Accessed November 19, 2021). 10.3389/fimmu.2021.744887 PMC8547269 34712232

[37] Chi X Yan R Zhang J Zhang G Zhang Y Hao M. A Neutralizing Human Antibody Binds to the N-Terminal Domain of the Spike Protein of SARS-CoV-2. Science (2020) 369:650–5. doi: 10.1126/science.abc6952 PMC7319273 32571838

[38] Yoshida S Ono C Hayashi H Fukumoto S Shiraishi S Tomono K. SARS-CoV-2-Induced Humoral Immunity Through B Cell Epitope Analysis in COVID-19 Infected Individuals. Sci Rep (2021) 11:5934. doi: 10.1038/s41598-021-85202-9 33723294 PMC7960719

[39] McCallum M De Marco A Lempp FA Tortorici MA Pinto D Walls AC. N-Terminal Domain Antigenic Mapping Reveals a Site of Vulnerability for SARS-CoV-2. Cell (2021) 184:2332–47.e16. doi: 10.1016/j.cell.2021.03.028 33761326 PMC7962585

[40] Suryadevara N Shrihari S Gilchuk P VanBlargan LA Binshtein E Zost SJ. Neutralizing and Protective Human Monoclonal Antibodies Recognizing the N-Terminal Domain of the SARS-CoV-2 Spike Protein. Cell (2021) 184:2316–31.e15. doi: 10.1016/j.cell.2021.03.029 33773105 PMC7962591

[41] Chen RE Zhang X Case JB Winkler ES Liu Y VanBlargan LA. Resistance of SARS-CoV-2 Variants to Neutralization by Monoclonal and Serum-Derived Polyclonal Antibodies. Nat Med (2021) 27:717–26. doi: 10.1038/s41591-021-01294-w PMC8058618 33664494

[42] Jangra S Ye C Rathnasinghe R Stadlbauer D Personalized Virology Initiative study group Krammer F. SARS-CoV-2 Spike E484K Mutation Reduces Antibody Neutralisation. Lancet Microbe (2021) 2:e283–4. doi: 10.1016/S2666-5247(21)00068-9 PMC8026167 33846703

[43] The Effect of Spike Mutations on SARS-CoV-2 Neutralization, in: Crick. Available at: https://www.crick.ac.uk/research/publications/the-effect-of-spike-mutations-on-sars-cov-2-neutralization (Accessed November 19, 2021). 10.1016/j.celrep.2021.108890 PMC7936541 33713594

[44] Weisblum Y Schmidt F Zhang F DaSilva J Poston D Lorenzi JC. Escape From Neutralizing Antibodies by SARS-CoV-2 Spike Protein Variants. eLife (2020) 9:e61312. doi: 10.7554/eLife.61312 33112236 PMC7723407

[45] Neutralization of SARS-CoV-2 Spike 69/70 Deletion, E484K and N501Y Variants by BNT162b2 Vaccine-Elicited Sera, in: Nature Medicine. Available at: https://www.nature.com/articles/s41591-021-01270-4 (Accessed November 19, 2021). 10.1038/s41591-021-01270-4 33558724

[46] Zani A Caccuri F Messali S Bonfanti C Caruso A. Serosurvey in BNT162b2 Vaccine-Elicited Neutralizing Antibodies Against Authentic B.1, B.1.1.7, B.1.351, B.1.525 and P.1 SARS-CoV-2 Variants. Emerg Microbes Infect (2021) 10:1241–3. doi: 10.1080/22221751.2021.1940305 PMC8216260 34092181

[47] Tauzin A Nayrac M Benlarbi M Gong SY Gasser R Beaudoin-Bussières G. A Single Dose of the SARS-CoV-2 Vaccine BNT162b2 Elicits Fc-Mediated Antibody Effector Functions and T Cell Responses. Cell Host Microbe (2021) 29:1137–50.e6. doi: 10.1016/j.chom.2021.06.001 34133950 PMC8175625

[48] Klingler J Lambert GS Itri V Liu S Bandres JC Enyindah-Asonye G. Detection of Antibody Responses Against SARS-CoV-2 in Plasma and Saliva From Vaccinated and Infected Individuals. Front Immunol (2021) 12:759688. doi: 10.3389/fimmu.2021.759688 34987505 PMC8721203

[49] Johnson S Bergthaler A Graw F Flatz L Bonilla WV Siegrist C-A. Protective Efficacy of Individual CD8+ T Cell Specificities in Chronic Viral Infection. J Immunol (2015) 194 (4):1755–62. doi: 10.4049/jimmunol.1401771 PMC4323683 25567678

