# Supplementary figures and images for: Natural SARS-CoV-2 Infection Affects Neutralizing Activity in Saliva of Vaccinees
Source: Front Immunol. 2022 Mar 11;13:820250. doi: 10.3389/fimmu.2022.820250 (PMC8962193; doi:10.3389/fimmu.2022.820250)

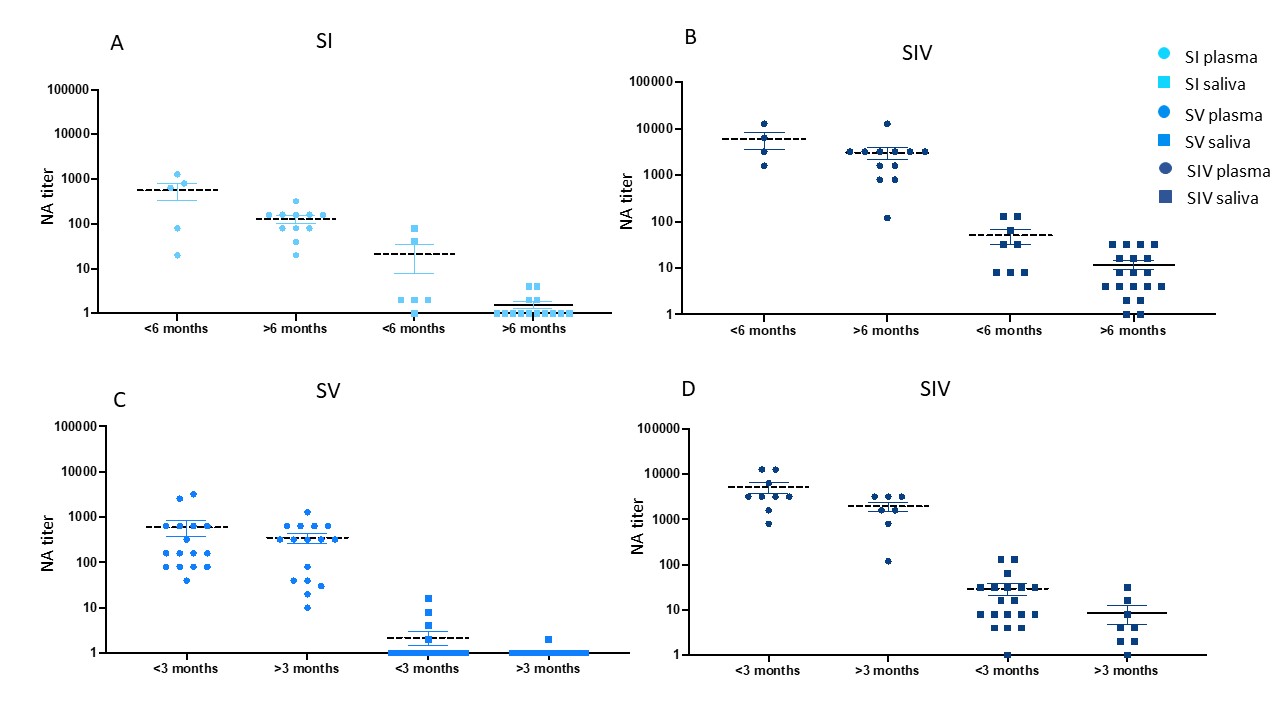

Supplement: Supplementary Figure 1 — Neutralizing activity (NA) from plasma and saliva of SARS-CoV-2 infected and/or vaccinated subjects, measured by Neutralisation assay (NTA) over time. NA in plasma and saliva samples of SI, and SIV analyzing according to the time from infection are reported in panel (A, B), respectively. (C, D) panels show NA in plasma and saliva of SV and SIV divided according to the time from vaccine administration. No statistically significant differences were observed in neither plasma nor saliva specimens from the enrolled groups over time. [file Image_1.jpg]

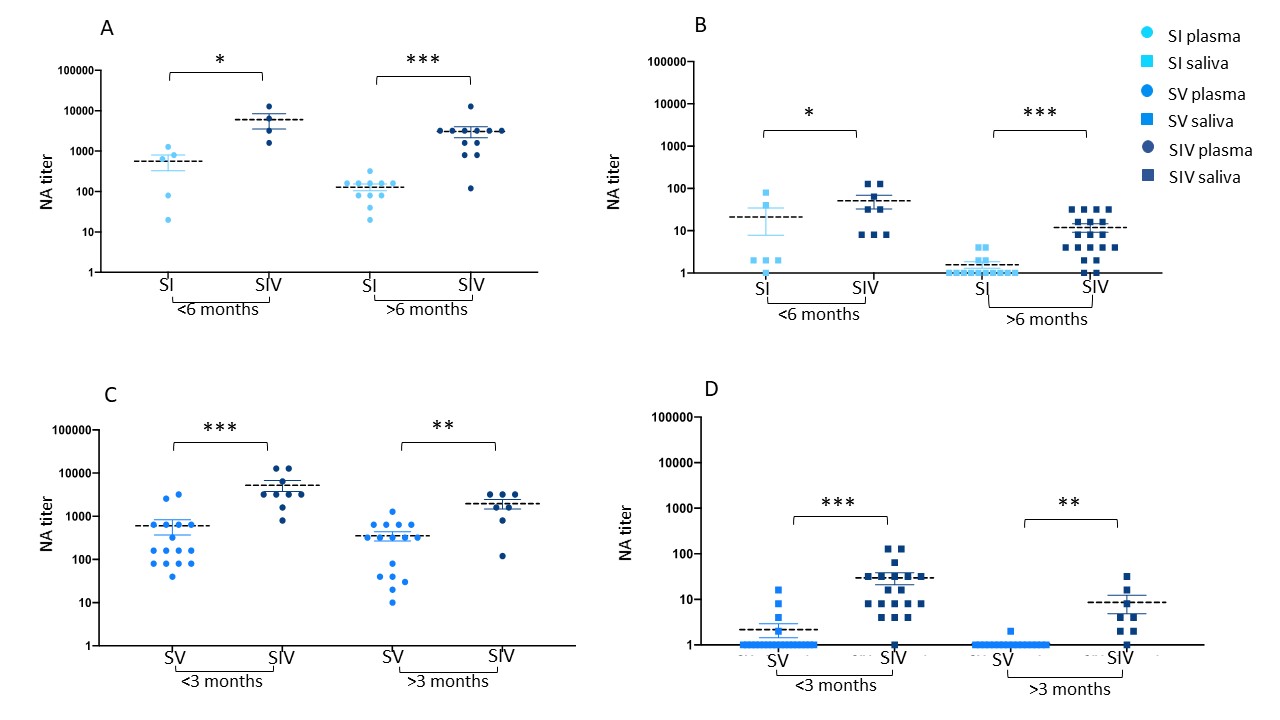

Supplement: Supplementary Figure 2 — Comparison of Neutralizing activity (NA) in plasma and saliva of subjects stratified according to the time from infection and/or vaccination. NA in plasma (A) and saliva (B) specimens from SI and SIV groups sampled before and after 6 months from symptoms onset. Plasma and saliva NA of SV and SIV groups are compared respectively in panel (C, D) before and after 3 months from vaccine administration. Significance difference are reported into the graph: *p<0.05, **p<0.01, ***p<0.001. [file Image_2.jpg]

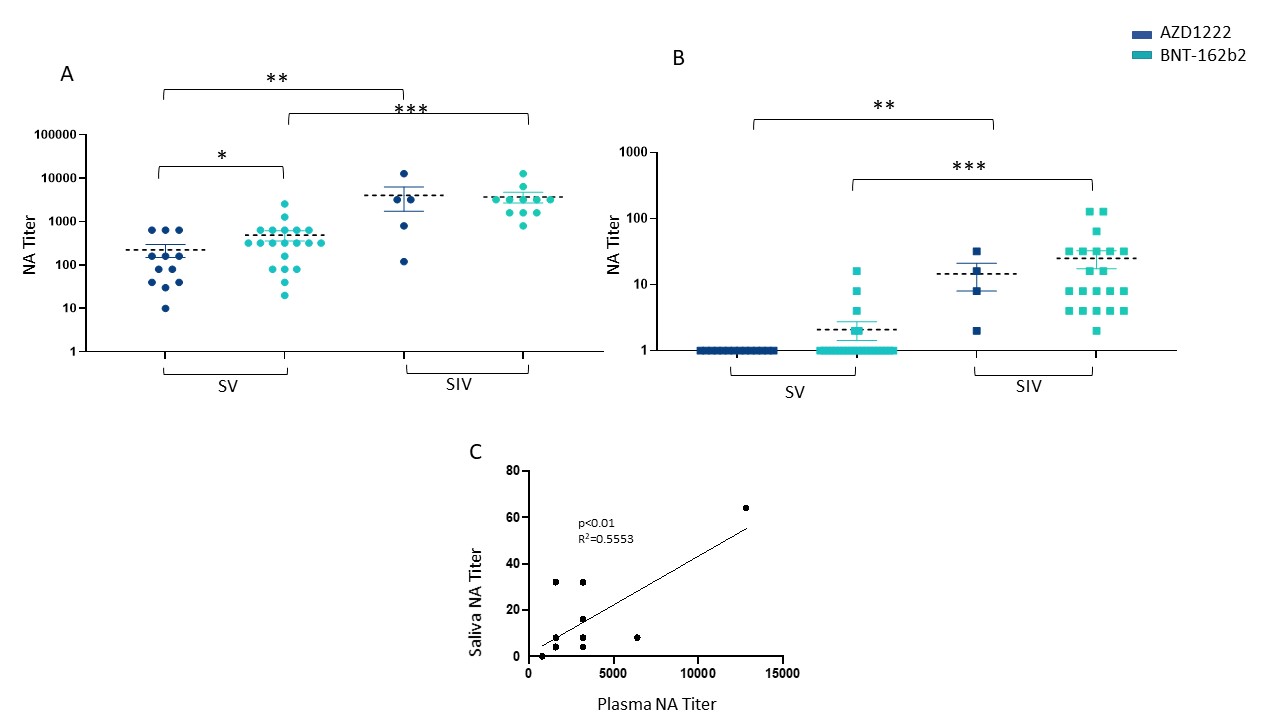

Supplement: Supplementary Figure 3 — Neutralizing activity (NA) in plasma and saliva samples of SV and SIV subjects, measured by virus neutralisation assay (vNTA). NA in plasma and saliva samples are reported in panel (A, B), respectively. Vaccinated subjects were divided according to the administrated vaccine: adenovirus-based (AZD1222) or mRNA (BNT162b2). *p<0.05, **p<0.01, ***p<0.001. In panel (C) the positive correlation between NA quantified in plasma and saliva samples from BNT-162b2 vaccinated subjects is shown. [file Image_3.jpg]

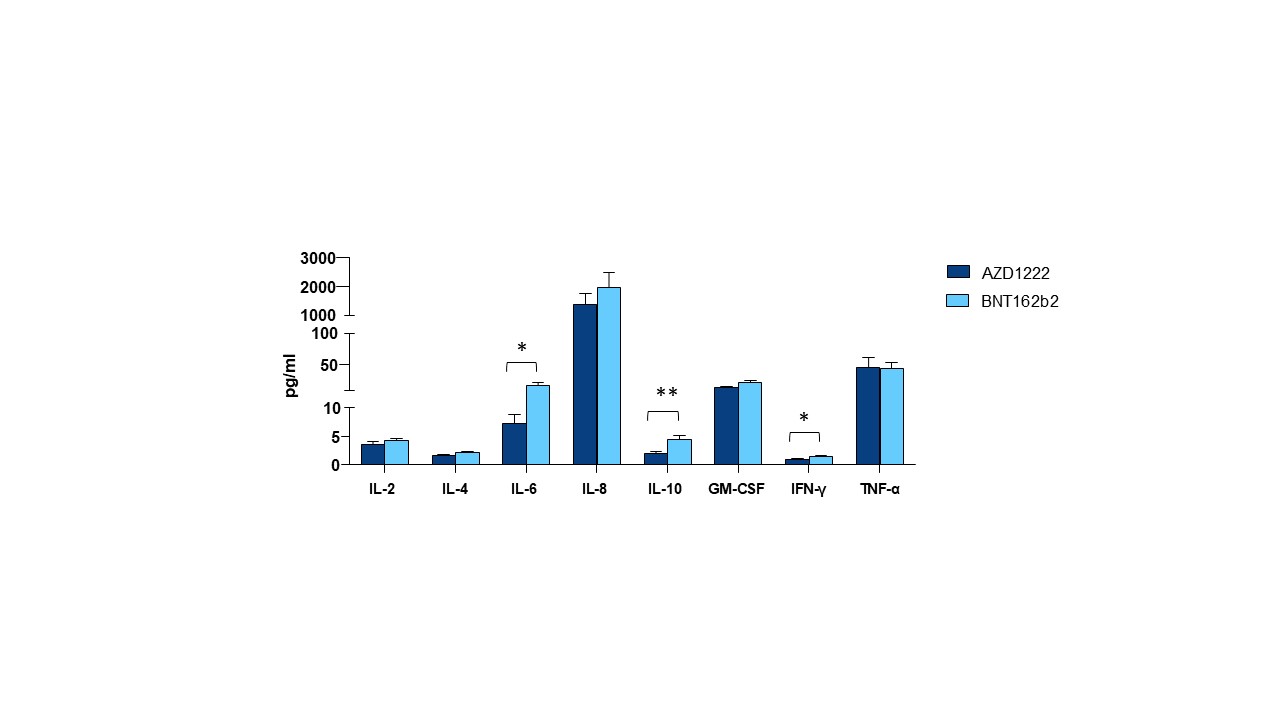

Supplement: Supplementary Figure 4 — Cytokine quantification in saliva samples from SV and SIV subjects. Cytokine concentration in saliva samples from SV (n=19) and SIV (n=21) subjects divided according to the anti-SARS-CoV-2 vaccine they were administered (AZD1222: n°=12; BNT162b2: n°=28). Mean values ± SE are reported. *p<0.05; **p<0.01. [file Image_4.jpg]
