# Supplementary material for: Natural SARS-CoV-2 Infection Affects Neutralizing Activity in Saliva of Vaccinees
Source: Front Immunol. 2022 Mar 11;13:820250. doi: 10.3389/fimmu.2022.820250 (PMC8962193; doi:10.3389/fimmu.2022.820250)
Supplement: Supplementary file 5 [file Table_1.pdf]

	subject Nº	iFlash SARS-CoV-2 IgG and IgM (YHLO)	
		Neg < 10 AU/mLPos > 10 AU/m	Neg < 10AU/mL Pos > 10AU/mL
		IgG	IgM
SV	1	0.1	0.3
	2	0.2	0.3
	3	0.1	0.2
	4	0.1	0.2
	5	0.3	2.1
	6	0.5	0.1
	7	0.2	0.1
	8	0.2	0.3
	9	0.1	0.4
	10	0.1	0.9
	11	0.1	0.1
	12	0.3	0.1
	13	0.6	2.2
	14	0.7	0.2
	15	0.1	0.3
	16	0.1	0.9
	17	0.2	0.9
	18	0.2	0.3
	19	0.3	0.9
	20	0.9	2.1
	21	2.1	0.5
	22	0.5	0.5
	23	0.5	2.0
	24	2.0	0.8
	25	0.8	0.1
	26	0.1	0.6
	27	0.6	0.2
	28	0.2	0.3
	29	0.3	0.6
	30	0.5	0.4
	31	0.2	1.9
	32	0.3	2.1

	33	0.2	1.5
	34	0.1	0.5
	35	2.3	1.0
	36	0.6	0.8
	37	0.4	2.1
	38	0.2	0.6
	39	0.2	0.2
	40	0.1	0.6
SI	1	16.3	0.3
	2	16.9	0.2
	3	20.6	0.1
	4	18.9	0.2
	5	23.3	0.1
	6	27.6	2.3
	7	29.6	2.2
	8	18.9	2.5
	9	22.6	2.6
	10	28.7	2.6
	11	18.8	3.9
	12	18.6	0.6
	13	17.1	4.5
	14	17.7	0.3
	15	28.5	2.6
	16	18.6	0.6
	17	30.3	0.1
	18	26.6	3.6
	19	21.6	2.1
	20	14.3	0.6
SIV	1	15.1	0.3
	2	25.6	3.6
	3	27.3	3.2
	4	17.3	0.1
	5	15.3	2.2
	6	14.9	0.1
	7	26.7	2.3

	8	12.3	2.2
	9	15.1	2.5
	10	20.6	2.6
	11	22.9	2.6
	12	29.8	0.9
	13	22.4	0.6
	14	16.3	4.5
	15	15.2	0.3
	16	21.6	1.6
	17	27.3	0.6
	18	20.0	0.1
	19	21.3	4.6
	20	22.6	2.1
	21	23.9	0.5
	22	22.2	6.3
	23	29.9	1.3
	24	21.6	2.5
	25	23.9	2.2
	26	23.6	1.6
	27	25.9	0.1
	28	17.0	0.2
